# Cerebral infarction by paradoxical gas embolism detected after laparoscopic partial hepatectomy with an insufflation management system: a case report

**DOI:** 10.1186/s40792-023-01611-0

**Published:** 2023-03-01

**Authors:** Kenji Shimizu, Masahiro Usuda, Yuta Kakizaki, Tomohiro Narita, On Suzuki, Kengo Fukuoka

**Affiliations:** Departments of Gastroenterological Surgery, Iwate Prefectural Central Hospital Morioka, Iwate, Japan

**Keywords:** Brain infarction, Laparoscopic hepatectomy, An insufflation management system, Patent foramen ovale

## Abstract

**Background:**

Laparoscopic surgery has reduced surgical morbidity and postoperative duration of hospital stay. Gas embolism is commonly known as a risk factor for all laparoscopic procedures. We report a case of severe cerebral infarction presumably caused by paradoxical CO_2_ embolism in laparoscopic partial hepatectomy with an insufflation management system.

**Case presentation:**

A male in his 60 s was diagnosed with recurrence of liver metastasis in the right hepatic lobe after laparoscopic lower anterior resection for rectal cancer. We performed laparoscopic partial hepatectomy with an AirSeal® under 10 mmHg of intra-abdominal pressure. During the surgery, the patient’s end-tidal CO_2_ and percutaneous oxygen saturation dropped from approximately 40–20 mmHg and 100–90%, respectively, while the heart rate increased from 60 to 120 beats/min; his blood pressure remained stable. Postoperatively, the patient developed right hemiplegia and aphasia. Brain magnetic resonance imaging showed cerebral infarction in the broad area of the left cerebral cortex. Thereafter, transesophageal echocardiography revealed a patent foramen ovale, suggesting cerebral infarction due to paradoxical gas embolism.

**Conclusions:**

A patent foramen ovale is found in approximately 15–20% of healthy individuals. While gas embolism is a rare complication of laparoscopic surgery, cerebral infarction must be considered a possible complication even if the intra-abdominal pressure is constant under 10 mmHg with an insufflation management system.

## Background

Laparoscopic surgery has led to a reduction in surgical morbidity, mortality, and length of postoperative hospital stay. Recently, laparoscopic hepatectomy has been recognized as a common surgical procedure for liver diseases. In laparoscopic procedures, carbon dioxide (CO_2_) is generally used for pneumoperitoneum; however, CO_2_ gas embolism is a serious complication in such cases [1, 2]. The incidence of gas embolism is rare approximately 0.15% in laparoscopic surgery, and 0.2–1.5% in laparoscopic hepatectomy [3], commonly caused by CO_2_ entrapment within an injured vein or solid organ. A serious complication is associated with paradoxical embolism, which occurs due to migration of the venous emboli into the arterial circulation through an arteriovenous shunt, resulting in cerebral infarction [4]. Herein, we present a case of cerebral infarction presumably caused by paradoxical embolism in laparoscopic hepatectomy.

## Case presentation

**Fig. 1:**
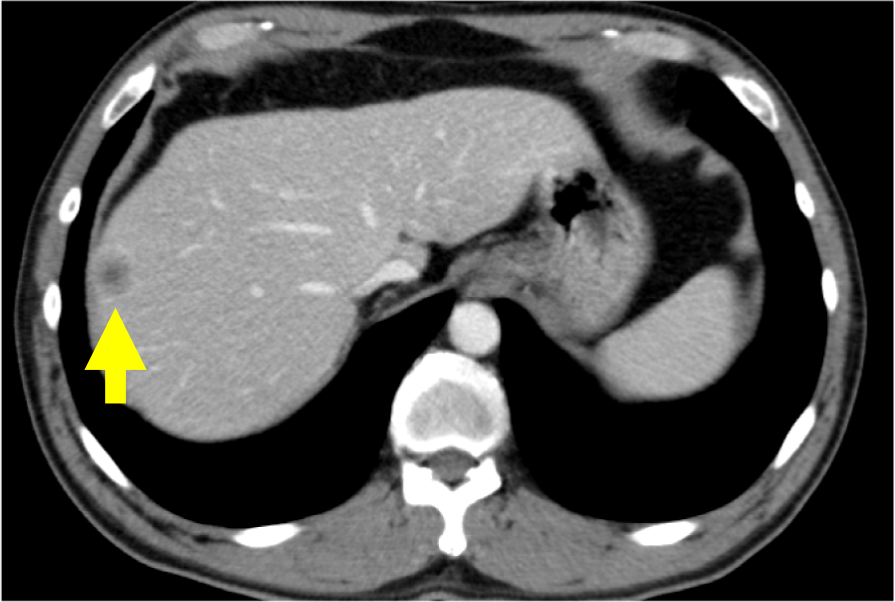
Contrast-enhanced CT. Contrast-enhanced CT showed a ring-enhanced tumor in the right hepatic lobe (arrow)

A male in his 60 s was diagnosed with recurrent rectal carcinoma of the right hepatic lobe on a regular Computed tomography (CT) examination and was scheduled for laparoscopic hepatectomy (Fig. 1). The patient had no clinical cardiopulmonary or brain diseases. Clinical examination revealed no relevant abnormal signs except for hyperlipidemia.

**Fig. 2:**
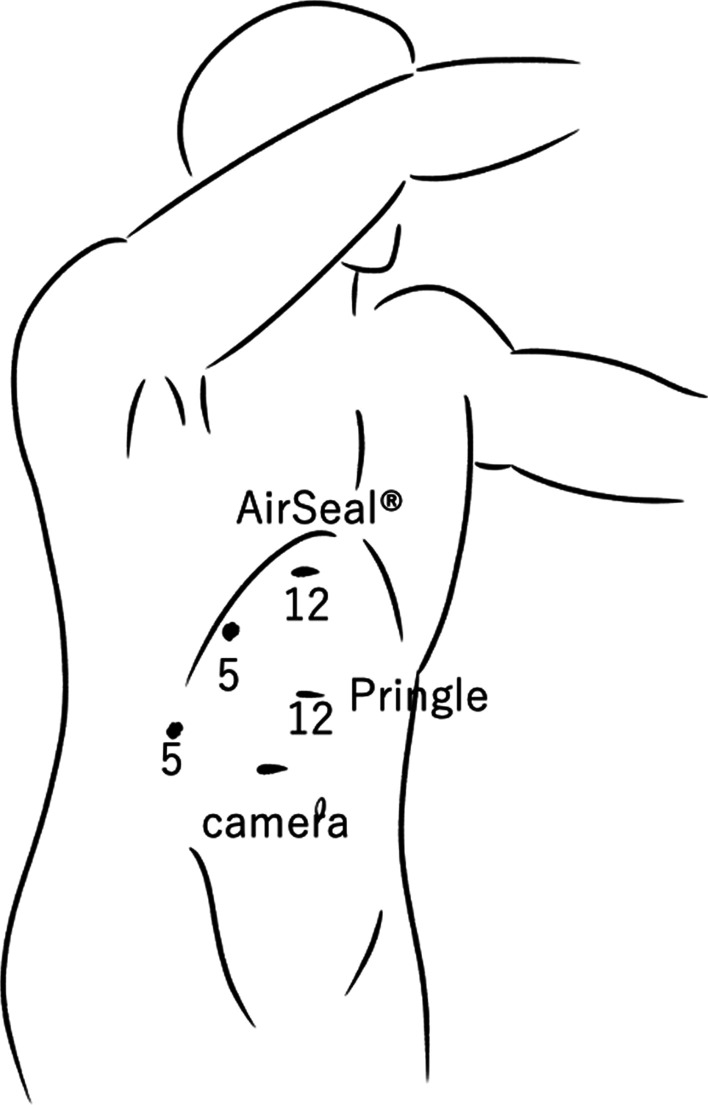
Patient and port positioning. The patient was placed in a head-up, left semi-lateral position. Two 12- and two 5-mm ports placed in the right and left paramedian, respectively. An AirSeal® trocar was placed through 12 mm of the cardiac fossa and manipulated by inserting right-hand forceps. Another 12 mm port was used for Pringle maneuver

**Fig. 3:**
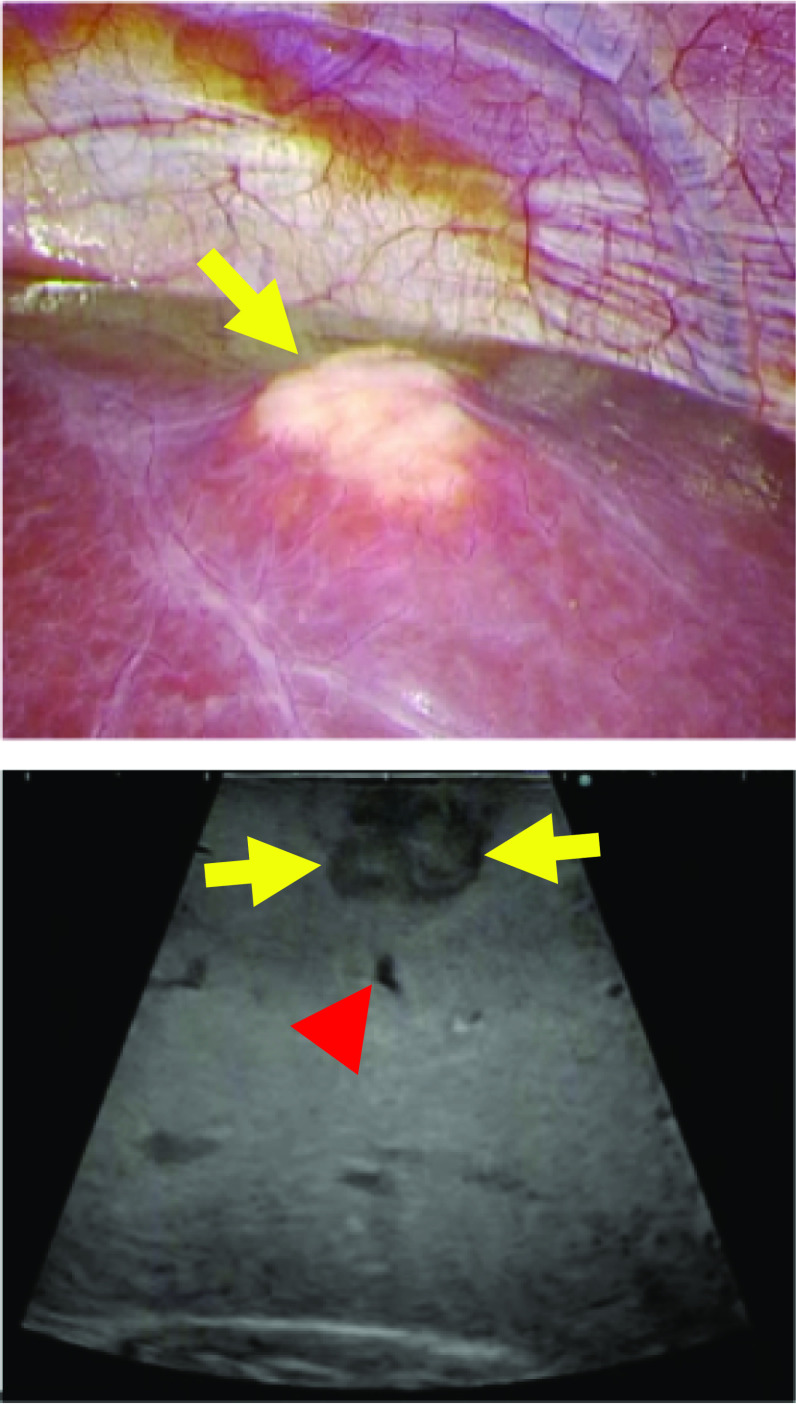
In the pre-operative work-up of patients with liver tumors. Laparoscopy prior to hepatectomy revealed that the tumor was located in segment 7 on the liver surface (Yellow arrow). Intraoperative ultrasonography confirmed metastasis in segment 7. It was 18 mm in size and distant from the main glissons, right hepatic vein, middle hepatic vein, and vena cava (Yellow arrow). There was no peripheral glissons at the base of the tumor and a peripheral hepatic vein 2 mm in diameter was identified (Red arrowhead)

**Fig. 4:**
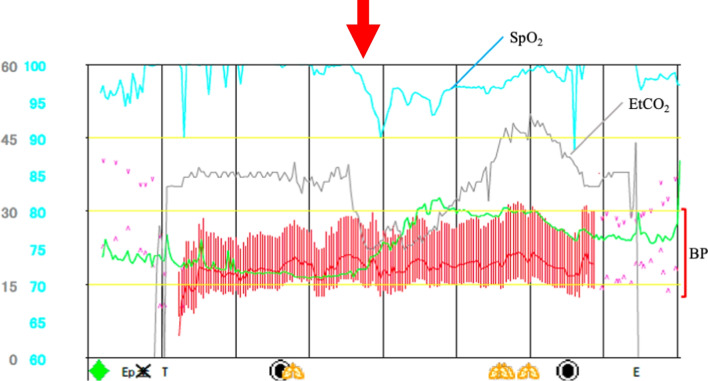
Anesthesia record. The patient’s EtCO_2_ dropped from 40 to 20 mmHg, percutaneous oxygen saturation decreased from 100% to 90%, and heart rate increased from 60 to 120 beats/min (arrow). SpO_2_: Saturation of percutaneous oxygen, EtCO_2_: End-tidal carbon dioxide, BP: Blood pressure

**Fig. 5:**
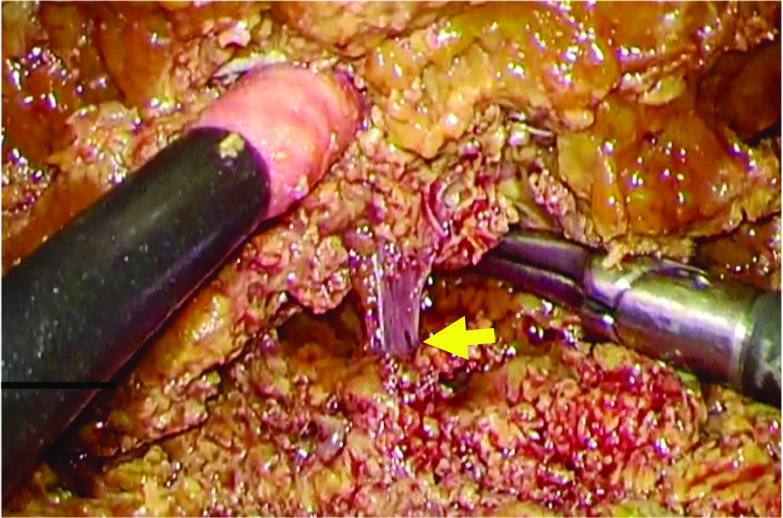
Laparoscopic image of the surgical field. A small laceration without hemorrhage was seen in the wall of the vein on the hepatic resection surface, which appeared to have been under the tumor (arrow)

The surgery, pure laparoscopic hepatectomy, was performed under combined general and epidural anesthesia. Pneumoperitoneum was performed using an insufflation management system (AirSeal iFs®; Conmed Corp., Utica, NY, USA) at low flow, with stable intra-abdominal pressure, starting at 8 mmHg and controlled up to 10 mmHg. The flow rate was 3 L/min and the smoke evacuation system retained low-level. During pneumoperitoneum, the tidal volume was adjusted to 500 mL, and the respiratory rate was maintained the end-tidal CO_2_ (EtCO_2_) at 30–40 mmHg. The fluid management strategy was controlled to obtain a positive end-expiratory pressure of 0 mmHg. A 12-mm trocar was inserted through right rectus abdominis as a camera port. An AirSeal® trocar was placed through 12 mm of the cardiac fossa and manipulated by inserting right-hand forceps. Another 12 mm port was used for Pringle maneuver. In addition, two 5-mm ports were placed in the right paramedian, respectively. The patient was placed in a head-up, left semilateral position (Fig. 2). Laparoscopy prior to hepatectomy revealed that the tumor was located in segment 7 on the liver surface, similar to the preoperative CT examination. Intraoperative ultrasonography confirmed metastasis in segment 7. It was 18 mm in size and distant from the main glissons, right hepatic vein, and vena cava. There was no peripheral glissons under the tumor and a peripheral hepatic vein 2 mm in diameter was identified (Fig. 3). We initiated laparoscopic hepatectomy after clamping the branches of the vascular pedicle, commonly known as the Pringle maneuver [5]. During liver parenchymal resection, we performed the clamp-crush method using Maryland forceps, or direct parenchymal division using scissors or vessel sealing system (LigaSure®, Medtronic, Minneapolis, USA). The small vessels were cut by the vessel sealer, small metal clips, or monopolar scissors. A laparosonic soft coagulation system (IO-Advance electrode®; AMCO Inc., Tokyo, Japan) was applied for transection of the liver parenchyma. At the first instance of the Pringle maneuver, the patient’s EtCO_2_ dropped from 40 to 20 mmHg, percutaneous oxygen saturation decreased from 100% to 90%, and heart rate increased from 60 to 120 beats/min. However, electrocardiogram showed sinus rhythm, and the blood pressure was almost stable at this time (Fig. 4). The surgery was continued without any special treatment, as the patient's hemodynamic state gradually improved before immediately deal with it. In the middle of the hepatic resection, a small laceration without bleeding was observed in the vein wall, which appeared to have been under the tumor (Fig. 5). The liver resection was completed without further complications. The tumors were extracted using a plastic bag through a camera-port incision. No drainage tubes were placed. The operative and pneumoperitoneum durations were 117 and 94 min, respectively. Intraoperative blood loss was minimal. The pathological specimen was about 3 cm in size, weighed 50 g, and the tumor was 18 × 15 mm.

**Fig. 6:**
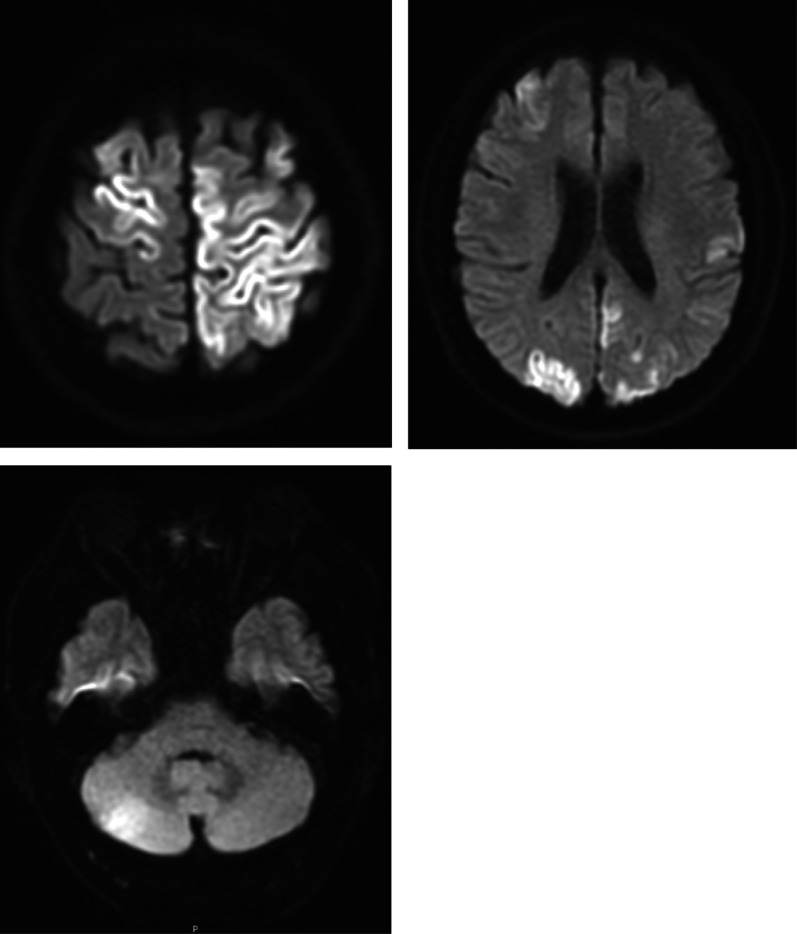
Postoperative T2-weighted magnetic resonance imaging of the brain. The yellow arrow indicates a high-density presence in the broad area of the left cerebral cortex

**Fig. 7:**
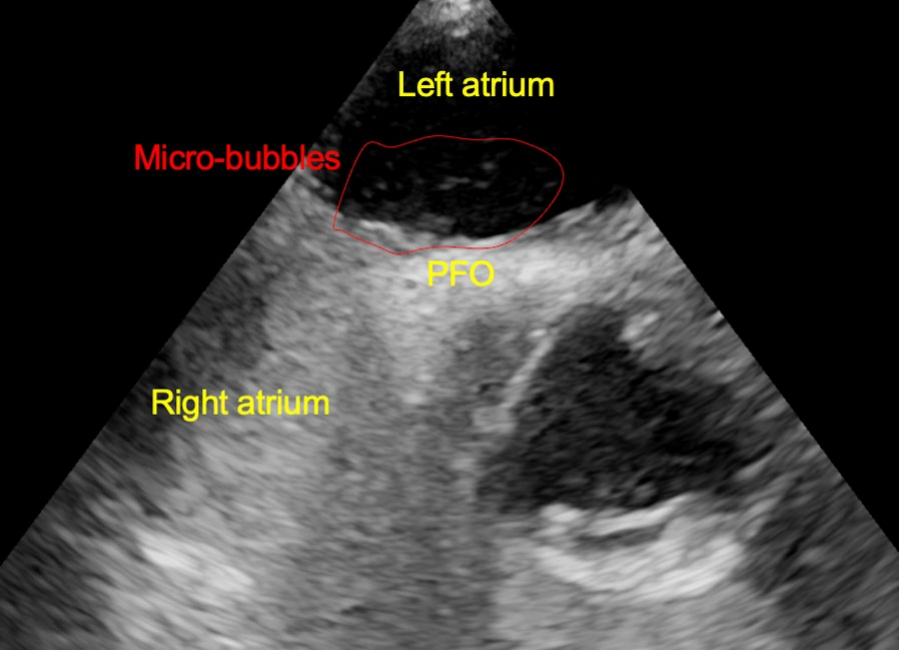
Postoperative transesophageal ultrasound examination affording two- and four-chamber long-axis views of the heart. A small atrial septal defect was seen (ovale) by micro bubble test

After surgery, the patient’s hemodynamic state was stable, and we smoothly performed tracheal extubation 20 min postoperatively. Although the patient was alert, he could not speak. He exhibited muscle weakness in the right arm and leg. Neurological examination at this point revealed a Glasgow Coma Scale score of 5/15 (E1V1M3). The patient breathed spontaneously, with eyes fixed toward the right with equal pupils, and had left motor palsy. Emergency magnetic resonance imaging (MRI) did not demonstrate any ischemic lesions. No obvious cerebral bleeding was detected on emergency CT postoperatively. The patient was then transferred to the intensive care unit for further observation. To minimize brain damage, we did not choose hypothermia therapy alternatively administered edaravone (3-methyl-1-phenyl-2-pyrazolin-5-one: free radical scavenger), for 2 weeks postoperatively. Follow-up MRI revealed cerebral infarction in the broad area of the left cerebral cortex on post operative day (POD) 1 (Fig. 6). To examine the cause of the cerebral infarction, we performed transthoracic echocardiography (TTE) on POD 2, which showed no thrombus or intracardiac defects. No atrial fibrillation was observed on electrocardiography. The patient was able to eat on POD 3. On POD 6, transesophageal echocardiography (TEE) revealed a patent foramen ovale (PFO) by micro bubble test [6, 7] (Fig. 7). Subsequently, he was screened by a neurologist for risk factors for stroke, including carotid ultrasonography, but no abnormalities were found. The patient was transferred to a rehabilitation hospital 21 days after the surgery. He was able to walk using a walking aid 6 months later and remained cancer recurrence-free at the 1-year follow-up.

## Discussion

Laparoscopic hepatectomy is performed worldwide. It is considered superior to open hepatectomy in terms of complication rate, duration of hospitalization, amount of hemorrhage, blood transfusion rate, and cost [8]. Hemorrhage is minimized for laparoscopic hepatectomy employing surgical techniques and generally anesthetic management [9–12]. However, gas embolism has been reported as a serious complication of this procedure [1, 2].

In terms of generally anesthetic management for hemorrhage control, the rate of intravenous fluid administration is maintained as low as 1 mL/kg/h, and the central venous pressure is controlled at ≤ 5 mmHg to facilitate the hemostatic effect of the pneumoperitoneum on the transection surface. During parenchymal transection, intra-abdominal pressure is maintained at 10–12 mmHg and raised to 15 mmHg in cases of massive bleeding not arising from the inferior vena cava. Under these circumstances, higher insufflation pressure than venous pressure may let injured vascular not to bleed resulting in leading to massive flow of insufflation gas into the vascular [12].

It is important to visualize the bleeding point in the surgical methods of hemorrhage control. In cases of bleeding, the point of bleeding is identified when suctioning the blood. Suction should be applied meticulously to avoid a reduction in intra-abdominal pressure. Low intra-abdominal pressure may cause a surge in bleeding, making hemostasis even more challenging and unachievable. Without proper care, suctioning is also a risk factor for gas embolization [13–15]. To prevent intra-abdominal collapse, a high-flow supply of CO_2_ immediately compensates for the pressure loss; the intra-abdominal pressure exceeds the venous pressure, and the gas is likely to flow into the vein. Thus, adequate central venous pressure should be maintained. Therefore, we should pay close attention to increase intra-abdominal pressure in laparoscopic hepatectomy.

According to a systematic literature review, three cases (0.2%) of gas embolism were reported among 1262 patients who underwent laparoscopic major hepatectomy (3). In comparison with general laparoscopic surgery, the incidence of CO_2_ embolism in laparoscopic hepatectomy (1.2–4.6%) was approximately 10 times of that in laparoscopic surgery (0.15%) [3, 16–18]. In the present case, no abnormalities regarding gas embolism were observed in a previous laparoscopic lower anterior resection for rectal cancer.

Gas embolism is a rare complication, and furthermore, cerebral infarction due to gas embolism could rarely occur [19]. In this case, we detected a PFO postoperatively, which is an intracardiac defect found in approximately 15–20% of healthy individuals [20]. The foramen ovale is usually closed under normal physiological conditions. It is possible that the right atrial pressure exceeded the left atrial pressure during surgery in this case, because PFO-opening and right-to-left shunting predominantly occur when the right atrial pressure exceeds the left atrial pressure. This likely caused a right-to-left shunt through the open PFO, secondary to a CO_2_ embolism, to develop into a cerebral infarction (Fig. 6).

Furthermore, we hypothesized other causes for the brain infarction in the present case. One of these causes could be the insufflation management system. This system enables surgeons to operate in a clearer field, as the automatic function constantly evacuates, filters, and recirculates gas back to its port [21]. This device maintains optimal insufflation not only by insufflating CO_2_ but also by entraining the room air, particularly throughout the sudden decrease in pressure that occurs in suctioning [22, 23]. Gas emboli using this device cannot be assumed to be “harmless” CO_2_ emboli, as insufflation gas may also contain air. Per a previous study, the abrupt introduction of 200 mL of air into the venous vasculature of an adult is lethal, whereas this amount is approximately equivalent to 1,000 mL CO_2_. The incidence of gas emboli during transanal total mesorectal excision surgery was estimated at 0.4% (25/6375 cases from reporting centers), but the insufflation system provided the presence of gas emboli in 24 of the 25 cases [14].

In this case, an AirSeal® trocar was placed through 12 mm of the cardiac fossa and manipulated by inserting right-hand forceps. Partial vascular injury was observed during hepatectomy. While hemorrhage was minimal due to pneumoperitoneum pressure, the trocar was oriented toward the injured vein, which might have facilitated gas embolization. However, these are only speculation and further evaluation such as in animal model experiments should be required.

Although symptoms such as decreased EtCO_2_ and SpO_2_, hemodynamic deterioration, electrocardiographic changes, and cardiac arrest are commonly observed in gas embolism, there were few cases of cardiopulmonary instability [24–26]. In laparoscopic hepatectomy before the use of AirSeal®, we had experienced several cases of CO_2_ embolization-like processes due to a drop in SpO_2_ and EtCO_2_. Even in any cases where blood pressure and other hemodynamic state were such that we had to stop surgery and wait for recovery, as we have never had any postoperative sequelae. In this case, there was a one-time decrease in EtCO_2_, SpO_2_, and an increase in heart rate, immediately after performing Pringle and starting liver resection. Because there were no other hemodynamic changes, we did not consider this case to be a special change. We did not consider the patient to have a serious gas embolism and continued the surgery without appropriate treatment. In retrospect, if we had taken appropriate measures at that time, this tragedy would not have happened. Because we never thought that there might contain air in his abdominal cavity.

To avoid the complications described herein, we should have examined the patient for an air embolism using TEE during surgery. TEE is considered the gold standard for diagnosis as it allows for direct anatomic visualization of shunting [27]. We could have quickly reduced the risk of paradoxical air embolism, if the TEE had detected the right atrium bubble in this case. Thereafter, we might have prevented cerebral infarction [28].

It is important to emphasize here that the three possible limiting conditions overlapped.

1. An unusual case in which the patient was asymptomatic and a PFO could not be recognized.
2. If a PFO had been known preoperatively, it would have been monitored intraoperatively with TEE.
3. The AirSeal®, which might not be a complete CO_2_ insufflator, had used as an insufflation device.

**Fig. 8:**
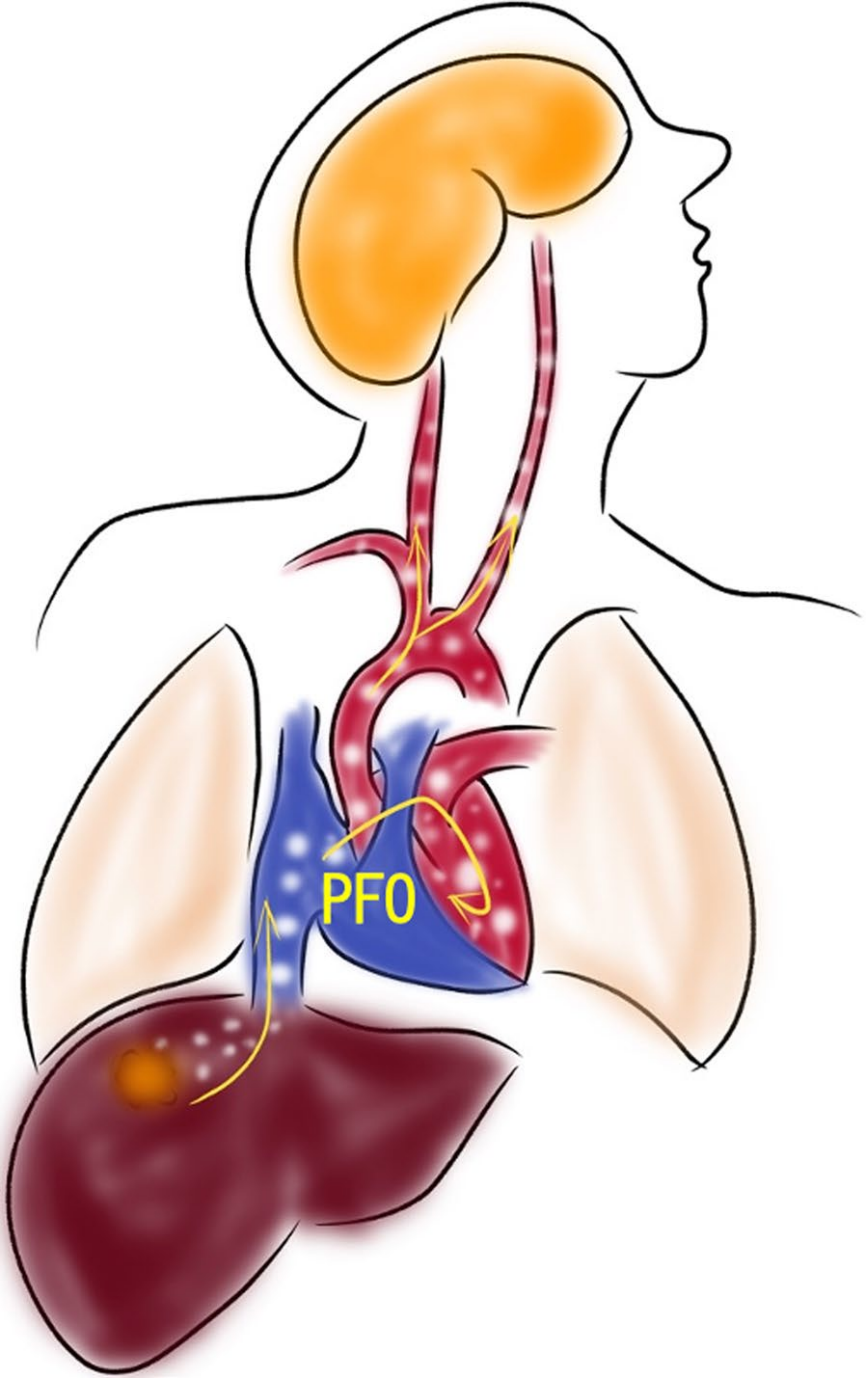
Schema of patent foramen ovale causes a right-to-left shunt. The shunt was secondary to a CO_2_ embolism, leading to cerebral infarction

In response to this, we have taken steps to ensure that this will never happen again, our hospital opened a morbidity and mortality conference in response to this case (Fig. 8).

1. Preoperative TTE is not usually performed to evaluate cardiac function in elderly patients or in patients without intolerance problems such as cardiovascular events. However, a microbubble test should be performed as a preoperative test for laparoscopic hepatectomy; if a PFO is present, it is mandatory to monitor it with intraoperative TEE.
2. Once EtCO_2_ and SpO_2_ drop, it should be assumed that a gas embolization has occurred, and measures such as pneumoperitoneum arrest and TEE monitoring should be promptly performed. If obvious hemodynamic instability is observed intraoperatively, including blood pressure and electrocardiogram, the operation should be terminated to stabilize the hemodynamic status. If this does not improve the patient's condition, a conversion procedure should be performed.
3. After this case, the use of AirSeal® in laparoscopic hepatectomy is contraindicated in our institution.

The relevant Japanese academic societies are talking about refraining from its use in laparoscopic hepatectomy. Although this case was treated with AirSeal®, because it was a case before such a report was made, we are awaiting the declaration of a ban on the use of this device in laparoscopic liver resection in the guidelines of the Japanese Society for Endoscopic Surgery.

## Conclusions

Cerebral infarction due to paradoxical gas embolism is a rare complication of laparoscopic surgery. However, the insufflation management system might have increased the risk of cerebral infarction. In addition, careful monitoring, such as that using TEE, and appropriate treatment for gas embolism, including cerebral infarction, are necessary in laparoscopic hepatectomy.

## Data Availability

All data generated or analyzed during this study are included in this published article.

## Abbreviations

CO_2_: Carbon dioxide
CT: Computed tomography
EtCO_2_: End-tidal CO_2_
MRI: Magnetic resonance imaging
PFO: Patent foramen ovale
POD: Post operative day
TEE: Transesophageal echocardiography
TTE: Transthoracic echocardiography
